# Breeding history and candidate genes responsible for black skin of Xichuan black-bone chicken

**DOI:** 10.1186/s12864-020-06900-8

**Published:** 2020-07-23

**Authors:** Donghua Li, Guirong Sun, Meng Zhang, Yanfang Cao, Chenxi Zhang, Yawei Fu, Fang Li, Guoxi Li, Ruirui Jiang, Ruili Han, Zhuanjian Li, Yanbin Wang, Yadong Tian, Xiaojun Liu, Wenting Li, Xiangtao Kang

**Affiliations:** 1 College of Animal Science and Veterinary Medicine, Henan Agricultural University, Zhengzhou, 450046 China; 2 Henan Innovative Engineering Research Center of Poultry Germplasm Resource, Zhengzhou, 450046 China; 3 The First Hospital, Jilin University, Changchun, 130062 Jilin China

**Keywords:** Xichuan black-bone chicken, Structural variants, Selective sweep, Black skin, Integration of whole genome and transcriptome

## Abstract

**Background:**

Domesticated chickens have a wide variety of phenotypes, in contrast with their wild progenitors. Unlike other chicken breeds, Xichuan black-bone chickens have blue-shelled eggs, and black meat, beaks, skin, bones, and legs. The breeding history and the economically important traits of this breed have not yet been explored at the genomic level. We therefore used whole genome resequencing to analyze the breeding history of the Xichuan black-bone chickens and to identify genes responsible for its unique phenotype.

**Results:**

Principal component and population structure analysis showed that Xichuan black-bone chicken is in a distinct clade apart from eight other breeds. Linkage disequilibrium analysis showed that the selection intensity of Xichuan black-bone chickens is higher than for other chicken breeds. The estimated time of divergence between the Xichuan black-bone chickens and other breeds is 2.89 ka years ago. *Fst* analysis identified a selective sweep that contains genes related to melanogenesis. This region is probably associated with the black skin of the Xichuan black-bone chickens and may be the product of long-term artificial selection. A combined analysis of genomic and transcriptomic data suggests that the candidate gene related to the black-bone trait, *EDN3*, might interact with the upstream ncRNA *LOC101747896* to generate black skin color during melanogenesis.

**Conclusions:**

These findings help explain the unique genetic and phenotypic characteristics of Xichuan black-bone chickens, and provide basic research data for studying melanin deposition in animals.

## Background

Domestication is a distinctive co-evolutionary, mutualistic relationship between humans and wild animals or plants that results in a range of genotypic and phenotypic impacts [1]. Animal domestication during the Neolithic period transformed the human lifestyle from hunting to farming, which enabled rapid changes in social organization and productivity [2]. Domesticated animals have spread to every region of the globe along with their human domesticators. The study of domestic animals contributes to our understanding of the evolution of animals under artificial selection, the influence of domestication or adaptive evolution on the animal genome, the genetic basis of evolution and phenotypic differentiation, and the optimization of animal breeding and diversity protection programs.

Chicken is one of the major sources of animal protein for humans. More recently, chickens have also become an important research model in fields such as physiology, disease, development, and aging, [3–6]. Chickens (*Gallus Gallus domesticus*) were the first domesticated bird species and were subjected for more than 8000 years to the combined effects of natural selection and human-driven artificial selection. Compared with their wild progenitors (red junglefowl, *Gallus gallus*), chickens present many characteristics associated with domestication that impact behavior, morphology, physiology, egg production, and skin color. A variety of studies have used whole-genome high-throughput DNA sequencing to reveal the genetic basis for traits acquired by natural and artificial selection in domesticated animals. For example, this approach has recently been applied to dogs [7], pigs [8, 9], chickens [10], sheep [11], rabbits [12], cattle [13, 14], and ducks [15, 16].

Studies suggest that northern China (alongside the Yellow River) is likely to have been one of several sites where chicken domestication occurred [17]. However, the history of breeding and the resulting phenotypic differentiation of indigenous chickens in this region have not been examined in sufficient detail. Both breeding history, and the genetic mechanisms underlying breed differentiation, have important theoretical implications for understanding the domestication, evolution, and phenotypic formation of chickens, and may also provide valuable insights for future breeding programs [18].

The Xichuan black-bone chicken (XBC), named for the Chinese prefecture of Xichuan, typically has five black parts (beak, skin, bones, legs, and meat) that distinguish it from other chicken breeds. Black-bone chickens are commonly believed to have medicinal properties and have been used as remedies to enhance the human immune system [19], prevent emaciation [20], treat diabetes [21], and cure conditions such as menstrual abnormalities and postpartum complications [22]. Xichuan black-bone chickens were primarily developed in a mountainous and inaccessible region in Xichuan County, China. Because transportation and trade were restricted, local resources were used for medical treatments, such as diet supplementation therapy. These incentives encouraged the selection of black skin in chickens. Xichuan black-bone chickens are highly prized, and became major income producers for farmers in the region. As demand increased, local farmers began to raise chickens at large scale and gradually developed this unique local chicken breed. However, the breeding history has not been examined in any detail, and the identity of the genetic factors responsible for the black body parts are unknown, particularly at the genomic level. In addition, the extent to which the Xichuan black-bone chickens has contributed germplasm to other breeds has not been studied.

In this study, we used whole genome resequencing to identify genomic features that illuminate the breeding history of the Xichuan black-bone chicken, and to correlate these features with the black characteristics of the breed. Genes with Xichuan black-bone chicken specific genomic variants were identified. Some genes in this class have undergone positive selection, and may be clues to the adaptive evolutionary history of the breed. By combining the genomic data with transcriptomic data, we were able to examine the genetic basis of adaptive evolution and breed differentiation more closely. Our results provide insight into the genetic factors underlying traits specific to Xichuan black-bone chickens. Furthermore, the data will provide a foundation for studying black coloration and modeling evolutionary selection mechanisms in this breed.

## Results

### Genetic variation in Xichuan black-bone chicken

**Fig. 1:**
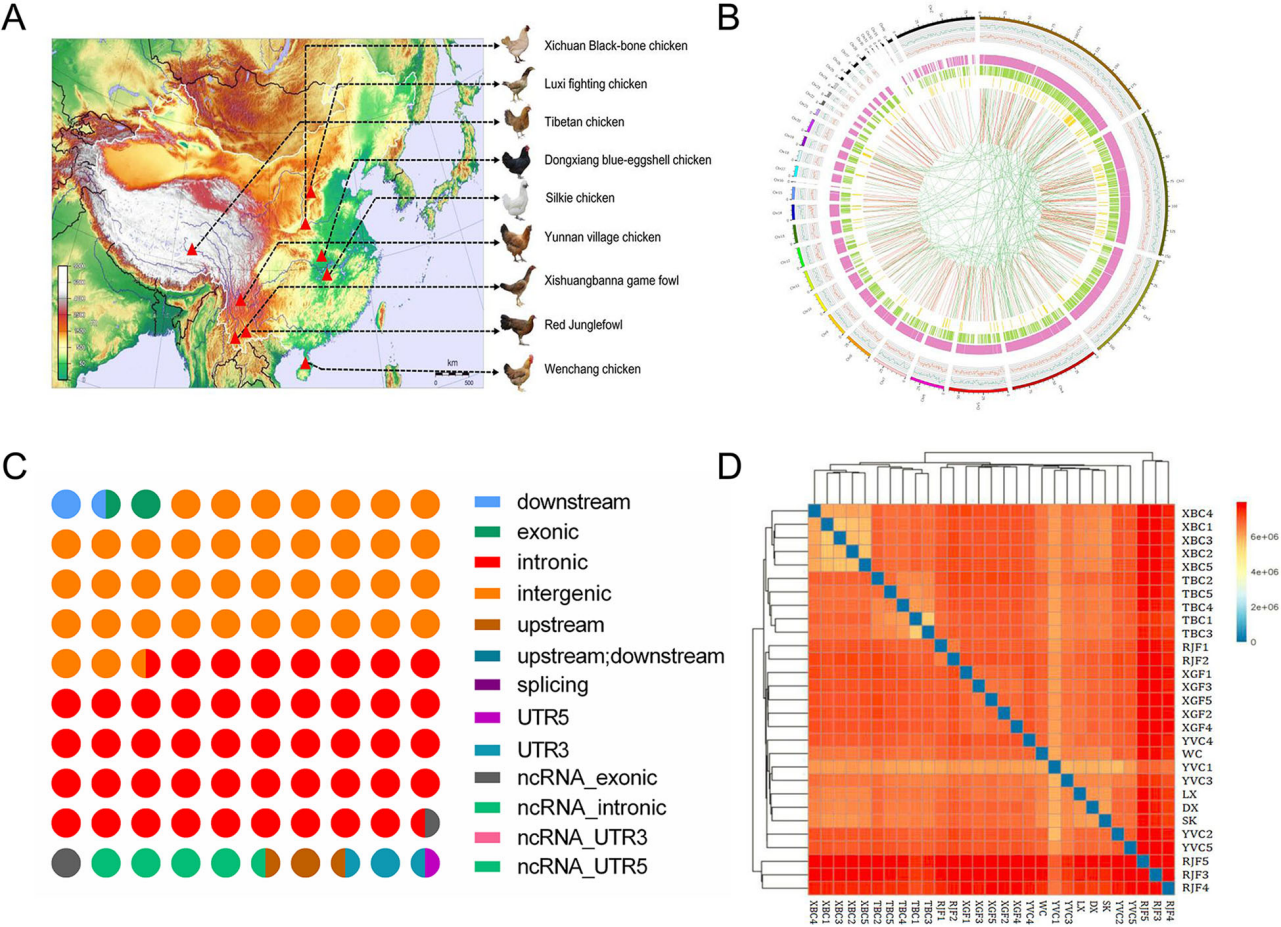
Experimental design and variant statistics. **a** Geographical origins of the 9 chicken breeds used in this study. The map was created by the Adobe Illustrator (AI) 2019 software (http://adobe.e-bridge.com.cn/shop/index.html). **b** Summary of genomic resequencing data from 5 Xichuan black-bone chickens. The figure shows the distribution of SNPs, indels, and SVs. Chromosomes are represented in different colors in the outermost circle. The remaining circles, in order from the outside to the inside, are as follows: genes, SNPs, indels, and SVs. **c** Distribution of SNPs based on context. Different colors represent SNPs within various functional regions. One circle represents 1% of total SNPs. **d** Heat map depicting genetic relationships based on SNP data obtained for the 29 chickens that represented the 9 breeds. Colors represent pairwise genetic distances

To identify genetic variations, we resequenced the genomes of 5 Xichuan black-bone chickens. Using the Illumina sequencing platform, each animal yielded over 213 million clean reads, representing approximately 32 Gb per chicken (Supplementary Table S4). Q30 scores exceeded 94%, and the average GC content was 43.89%. The average sequencing depth was 28-fold per individual (a total of 160.90 Gb of high-quality paired-end sequence data). Around 98.45% of Xichuan black-bone chicken sequences were identical to those of red jungle fowl. The gene density per 200 kb and the number of SNPs, insertion/deletion polymorphisms (InDels), CNVs and SVs per 100 kb are shown in Fig. 1b. We identified 5,062,529 SNPs, including 247,054 homozygous SNPs, 830,606 InDels (372,903 insertions and 457,703 deletions), 1279 CNVs, and 11,433 SVs (Supplementary Fig. S1).

To better understand the distribution of SNPs, we classified them according to their context into 13 categories (Fig. 1c). 39.23% of SNPs were found in intergenic regions. An even larger number were located in introns (47.23%). Smaller numbers of SNPs were within 5′ and 3′ UTRs (2.55% in aggregate), upstream or downstream of genes (3.14% in aggregate), splicing regions (0.01%), or were associated with ncRNA genes (6.88% in aggregate). Moreover, IS analysis indicated that chickens from the same habitat were more likely to have similar genetic distance and the clearest clusters (Fig. 1d).

### Population structure and domestication

**Fig. 2:**
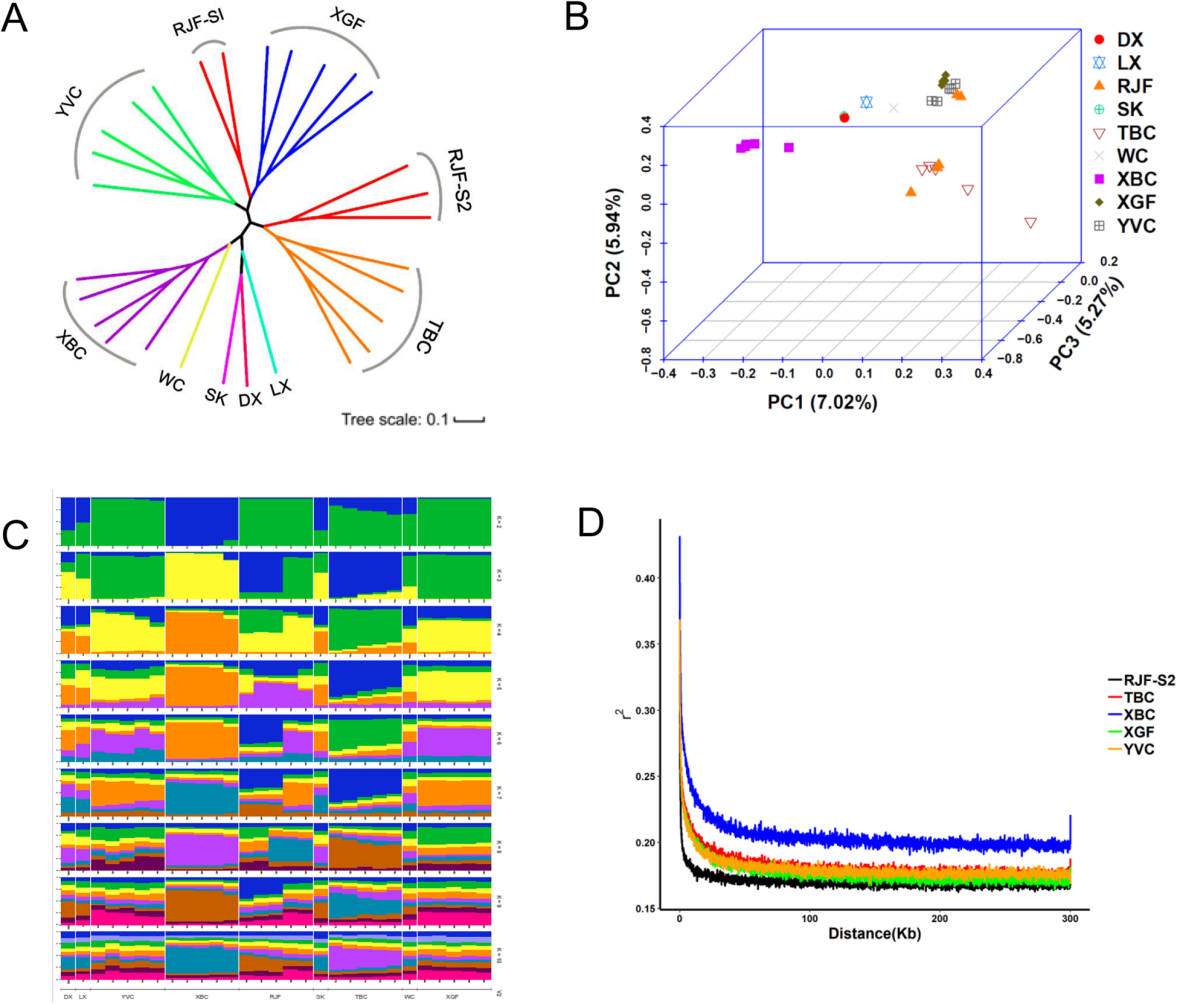
Population genetics and LD decay. **a** NJ tree generated using polymorphisms detected in the 29 individual chickens. The scale bar represents the evolutionary distances measured by p-distance. Each of the 9 breeds has been assigned a distinct color. **b** Three-way PCA plots based on the 29 chickens. Symbol colors indicate breed (key on right). **c** Genetic structure of samples from 29 individuals for K groups using the ADMIXTURE program. K is the number of presumed ancestral groups, which was varied in the analysis from 2 to 10. The optimal K value was obtained with the least CV error value. **d** Decay of LD for XGF, YVC, RJF, TBC and XBC chickens measured by R^2^

To examine genome-wide relationships and divergence between Xichuan black-bone chickens and other populations, we constructed a NJ tree with 1000 bootstrap replicates based on whole-genome polymorphic SNPs (Fig. 2a). The chickens clustered into three major branches that reflect geographic origins and breed. While YVC formed a distinct clade, the 5 RJFs fell into two clades (RJF-S1 and RJF-S2) that associated with other chickens (RJF-S1 and XGF, and RJF-S2 and TBC). This result aligns with their geographical distributions rather than their breed ascription, and is consistent with previous studies [23, 24], reinforcing the hypothesis that RJF was domesticated in several areas, as suggested by an analysis of mtDNA sequences [25, 26]. XBC clustered with WC, SK, DX, and LX, within the same branch. Results obtained using PCA were consistent with the NJ tree. The first three principal components accounted for 7.02% (PC1), 5.94% (PC2), and 5.27% (PC3) of total variability. The breeds formed distinct groupings except for RJF (Fig. 2b).

Figure 2c shows a structure plot representing the 29 sampled chickens. At a low value of K (K = 2), XBC is clearly associated with a separate ancestor. When K was set to 4, 2 individuals from RJF, YVC and XGF (i.e., six individuals total) presented similar major components, while XBC and TBC appeared to cluster separately. XBC became almost distinguishable from all other breeds with increasing values of K. To estimate LD in the XGF, YVC, RJF, TBC, and XBC breeds, we calculated the squared correlations for two loci against the genome distance R^2^ between pairs of SNPs. Because the NJ tree analysis and PCA showed RJF-S2 to be relatively independent, we used RJF-S2 for the LD analysis. As shown in Fig. 2d, the most rapid attenuation was observed in RJF, followed by YVC, XGF, TBC, and XBC (Supplementary Table S5), indicating these breeds have relatively higher diversity and lower selection intensity. In contrast, the slowest attenuation was found in XBC. Thus, the XBC has experienced more domestication and selection intensity than have the other breeds.

### Analysis of gene flow, time of divergence, and demographic history

**Fig. 3:**
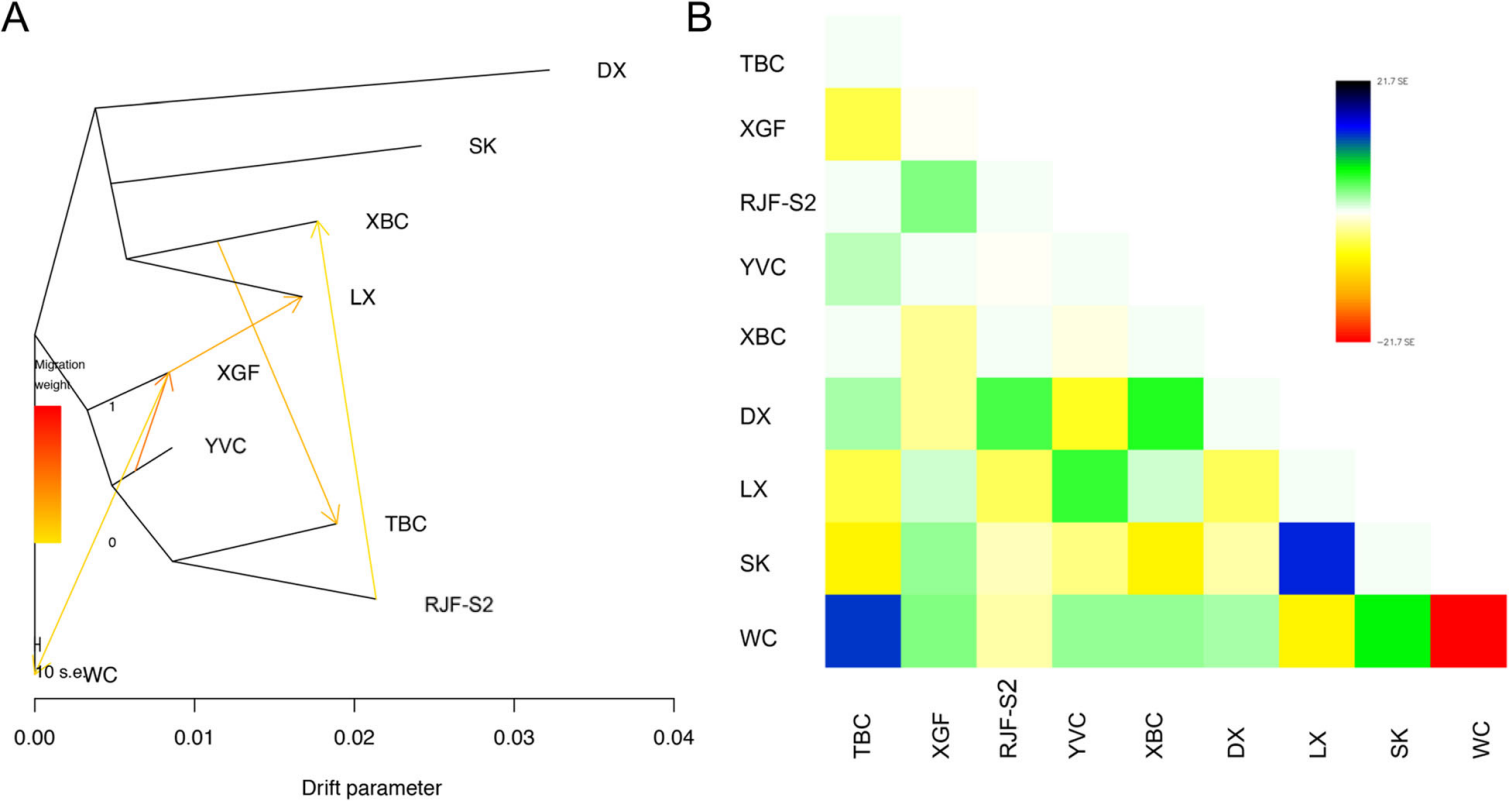
Gene flow analysis. **a** Maximum likelihood tree with 5 migration events. Migration events are shown as colored arrows, shaded according to their weight. Horizontal branch lengths are proportional to the amount of genetic drift that has occurred on each branch. The scale bar shows 10 times the average standard error of the entries in the sample covariance matrix. **b** Residual fit from the maximum likelihood tree in (**a**)

We used TreeMix [27] to examine the topology of relationships and migration history among populations. We observed an early split between western (TBC), central (DX, LX, SK and XBC), and southern (XGF and YVC) populations (Fig. 3a & Fig. 3b). We detected a genetic contribution from RJF-S2 to XBC, and also found gene flow between XGF and LX, both of which are raised for cockfighting. Interestingly, a gene flow was observed from XBC to TBC (Fig. 3a & Supplementary Fig. S2).

**Fig. 4:**
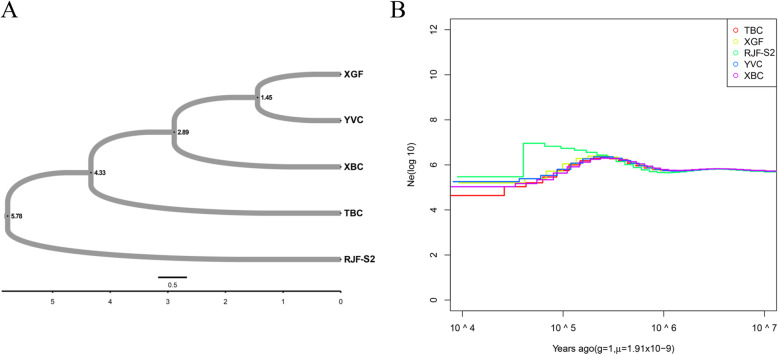
Population genetics and demographic history. **a** Time of divergence between populations. The number at each node represents the time of divergence in thousands of years. **b** Demographic history of XBC and 4 other Chinese chicken breeds. Generation time (g) = 1 year and trans-version mutation rate (μ) = 1.91 × 10^− 9^ mutations per base pair per generation

XBC differentiation time was analyzed by combining the available literature and fossil archeological data [17, 26, 28]. As shown in Fig. 4a (Supplementary Table S6), we found that XGF is much more closely related to YVC than to other chickens. The estimated time of divergence between XGF and YVC is 1.45 ka years ago, while the estimated divergence time of XBC is 2.89 ka years ago. Moreover, the results also demonstrated that the earliest differentiation occurred in RJF-S2 (5.78 ka years ago).

As the unique genetic characteristics of XBC might be related to distinct divergence events, we conducted a PSMC for XBC and other Chinese populations to infer historical changes in effective population size (Ne). A tendency toward increased population size was detected in five of the populations 5 ka years ago (Fig. 4b), reaching a peak at the Last Glacial Maximum (20–26.5 ka), with a dramatic decrease following that peak [29]. The effective population size declined after the Last Glacial Maximum, with the increase in global temperature and development of human civilization [30].

### Genome-wide selective sweep signals and functional analysis

**Fig. 5:**
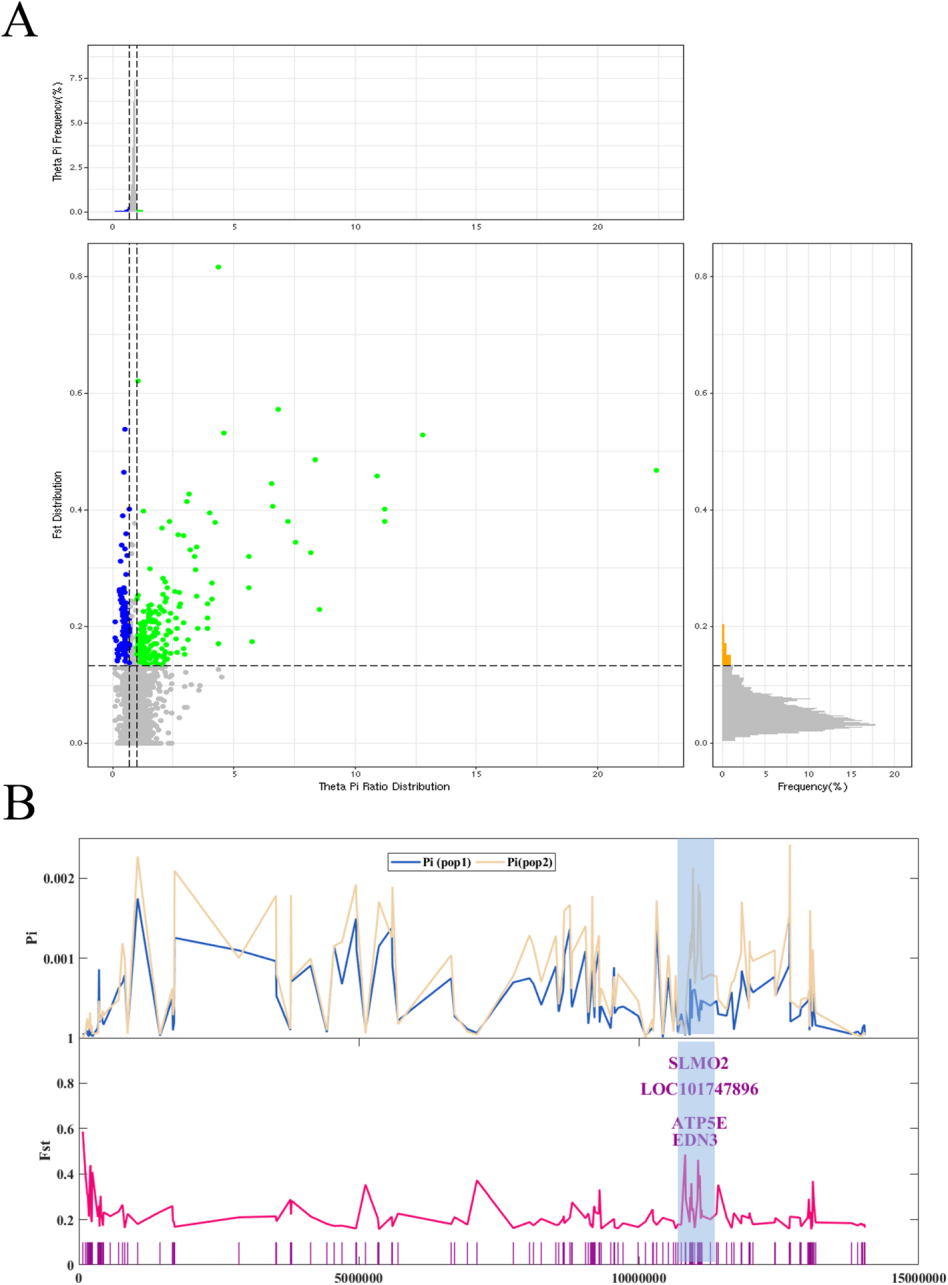
Identification of genomic regions in Xichuan black-bone chickens with strong selective sweep signals. **a** Selective sweep signals are located to the left and right of the vertical dashed lines (representing Pi ratio values > 0.95 or < 0.05, respectively), and above the horizontal dashed line (representing an *Fst* value > 0.17). Regions selected for black skin are shown using blue points, while other skin colors are shown in green. The x-axis shows the pi ratio between black-skin and non-black-skin groups, and the y-axis shows the *Fst* values. **b** Genes with selective sweep signals in black and non-black skin. The shaded genomic regions contain selective signals for both skin types. pop1 and pop2 represent black skin non-black skin, respectively

**Fig. 6:**
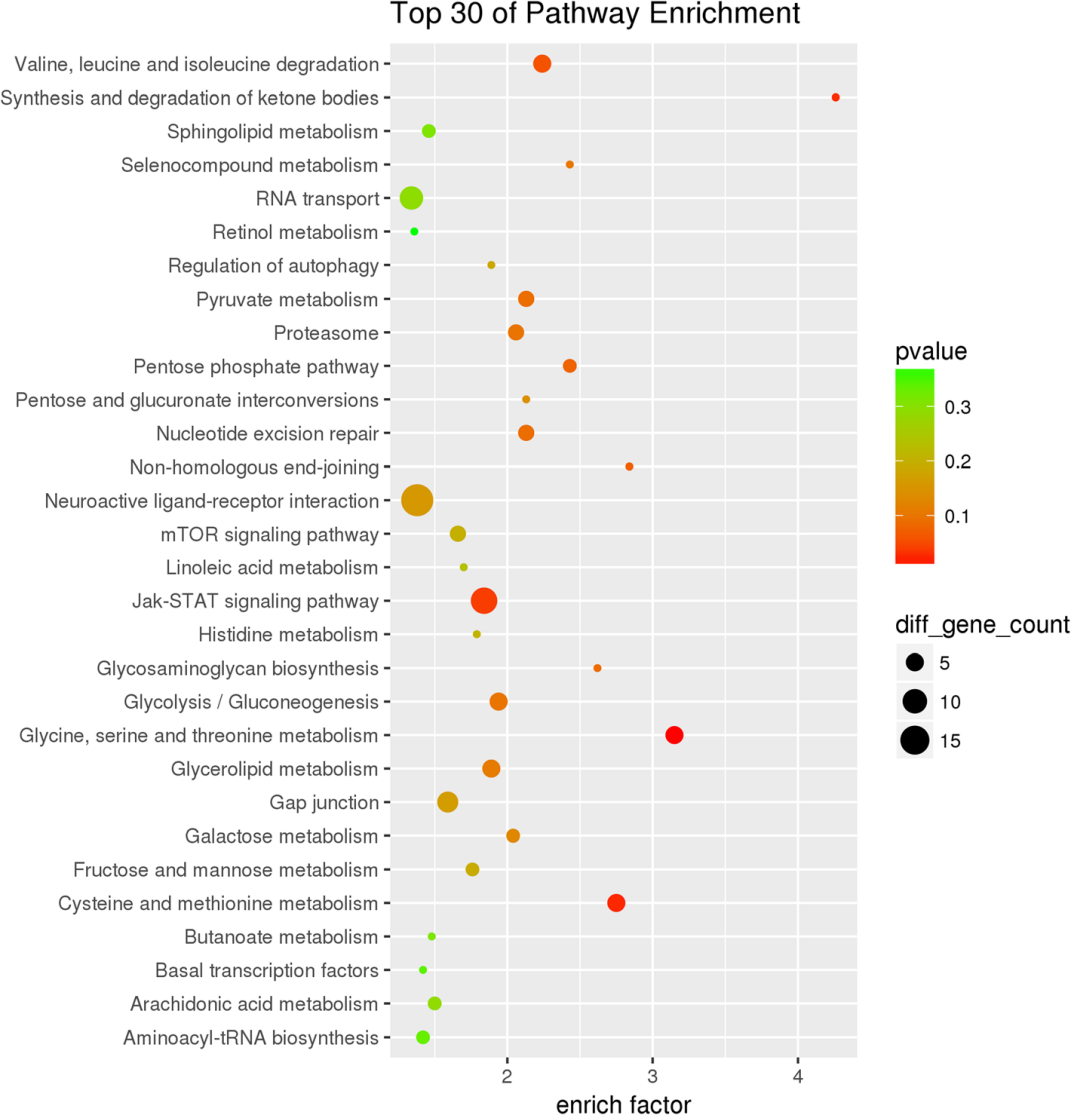
KEGG pathway enrichment analysis of candidate genes under selection in black skin chickens

In order to better detect genome-wide selection signals related to the unique black-skin trait, we divided the populations into black-skin and non-black-skin groups. Both the pi cut-off ratio (top 5%, pi ratio > 0.95 or < 0.05) and high *Fst* values (top 5%, *Fst* value> 0.17) were used as criteria for classifying selective sweeps. A total of 1469 candidate genes within these sweeps were associated with black-skin (Fig. 5a & Supplementary Table S7). Among them, one sweep located on chromosome 20 exhibited a high *Fst* value, indicating obvious genetic differentiation between black-skin and non-black-skin populations. In addition, a large difference in the pi value was also observed within this sweep between black and non-black groups. The pi value in the black-skin group was lower, suggesting that the sweep has been positively selected in the black-skin population (Fig. 5b). The candidate genes were then subjected to functional analysis. Top 30 of GO analysis revealed 10 GO terms enriched in biological processes, 8 terms in molecular functions, and 12 in cellular components (Supplementary Fig. S3). The KEGG pathway analysis results showed that candidate genes are mainly involved in the neuroactive ligand-receptor interaction, Jak-STAT signaling pathway, and so on (Fig. 6).

### Candidate genes for skin pigmentation

Skin color is an important domestication trait in chickens. We analyzed differences between black-bone chickens and other domesticated chickens to detect selection signatures associated with black skin. Genes associated with pigmentation that play important roles in the regulation of melanin deposition in mammals were identified in several genomic regions within selective sweeps. The candidate list includes solute carrier family 45 member 2 (*SLC45A2)*, oxysterol binding protein like 2 (*OSBPL2*),

solute carrier family 24 member 2 (*SLC24A2*), PRELI domain containing 3B (*SLMO2*), ATP synthase, H+ transporting, mitochondrial F1 complex, epsilon subunit (*ATP5e*), cyclin dependent kinase inhibitor 2A (*CDKN2A*)*,* GRAM domain containing 3 (*GRAMD3*), fibroblast growth factor 10 (*FGF10*) and Endothelin3 (*EDN3*).

**Fig. 7:**
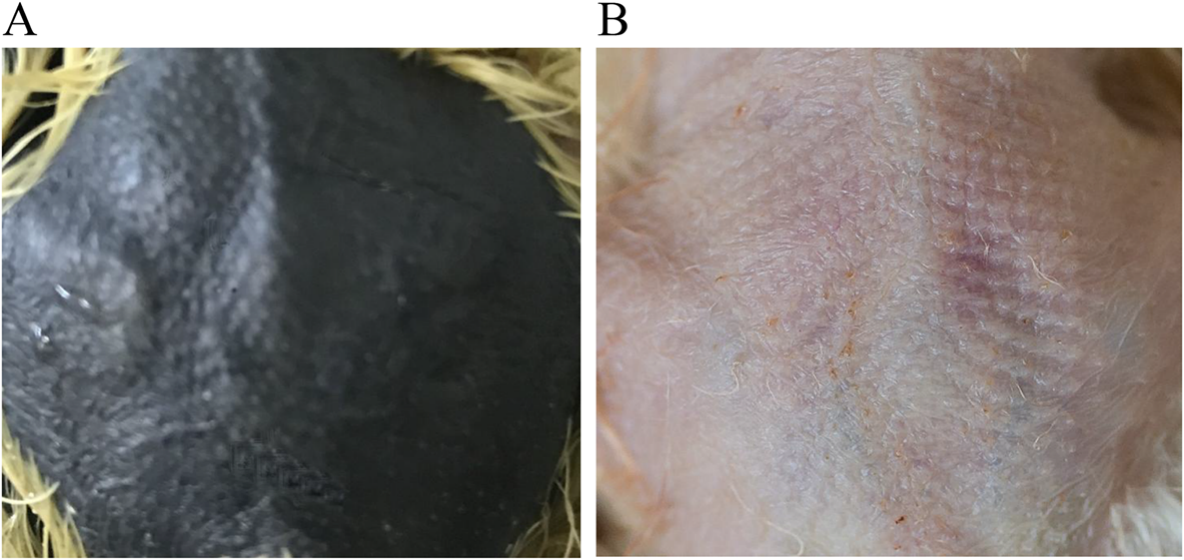
Histomorphological examination of Xichuan black-bone chickens with black (**a**) and yellow (**b**) skin. The picture was taken by Canon camera (5D Mark IV)

**Fig. 8:**
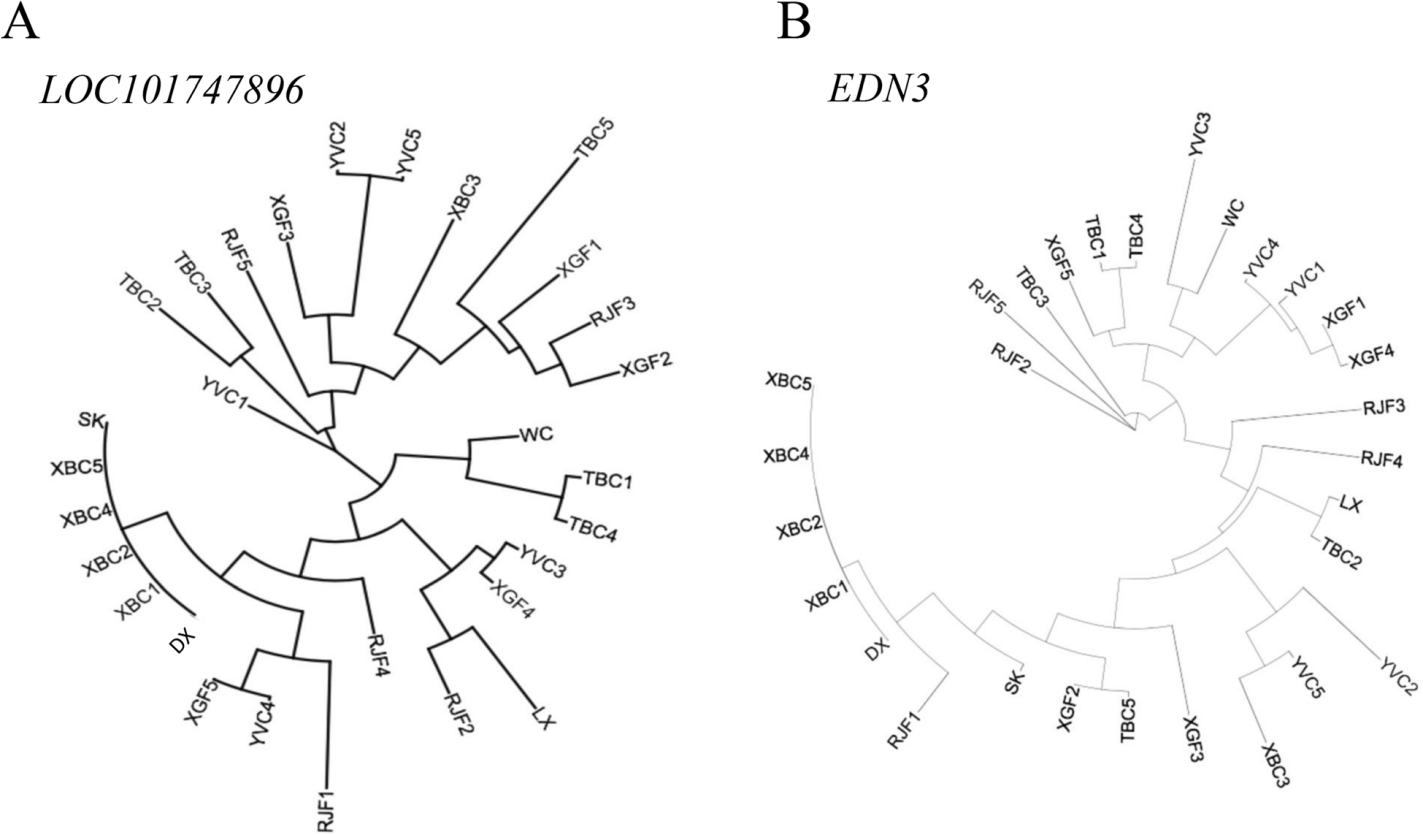
Gene trees for *LOC101747896* (**a**) and *EDN3* (**b**), based on 29 chickens

To better understand the evolution of the selected genes, we hybridized Gushi chickens with Xichuan black-bone chickens to obtain F2 full-sibs with different skin colors. Yellow-skin and black-skin individuals with the same genetic background were then used for RNA-seq (Fig. 7). Four selected genes (*SLC45A2, SLMO2, ATP5e*, and *EDN3*) were differentially expressed between the black and yellow skin groups (Supplementary Table S8). A differentially expressed long noncoding RNA (*TCONS-00054154*) was also identified that is potentially related to black skin (Supplementary Table S9). *TCONS-00054154* was used as a query using NCBI BLAST to identify related sequences, and was found to be identical to *Gallus gallus* uncharacterized LOC101747896, transcript variant X5, ncRNA. Both ncRNAs are located near *EDN3*, *SLMO2* and *ATP5e* (Supplementary Table S10), suggesting that *TCONS-00054154* might function in regulating these genes to produce different skin colors during melanogenesis. NJ analysis of *EDN3* and *TCONS-00054154* showed that they cluster to one branch in black-skin chickens (SK, DX, and XBC) (Fig. 8). This supports the hypothesis that these two genes might interact with the candidate genes and affect pigmentation in black skin.

Among the candidate genes, we found that fatty acid desaturase 6 (*FADS6*), Leptin receptor (*LEPR*), Lipoprotein lipase (*LPL*), melanin-concentrating hormone receptor 1 (*MCHR1*) and perilipin 2 (*PLIN2*) were associated with fatty acid-related pathways. Additionally, solute carrier organic anion transporter family member 1A2 (*SLCO1A2*), solute carrier organic anion transporter family member 1B1 (*SLCO1B1*), and solute carrier organic anion transporter family member 1C1 (*SLCO1C1*) belong to the organic ion transporter polypeptide (OATP) gene family, and may affect the transport of pigment in eggshells.

### Identification of differentially expressed genes by qRT-PCR

As shown in Supplementary Fig. S4, gene expression levels were determined by RNA-seq sequencing and qRT-PCR. Our result suggested that the expression level for six genes obtained by qPCR were consistent with the high-throughput data, which showed the reliability of RNA-seq data.

## Discussion

Xichuan black-bone chickens are rare in China and elsewhere, and have not been the focus for many studies. Due to the limited information available, we performed in-depth whole-genome sequencing of 5 black-bone chickens to explore the population structure, genetic diversity, and history of this breed. The data were analyzed along with sequences for other Chinese breeds that were downloaded from NCBI.

Sequence variations were distributed across different chromosome in numbers roughly proportional to chromosome size. Using the variations as the basis for our analysis, we found that Chinese chickens are divided into three large groups. Chickens from neighboring geographical locations and from similar altitudes are gathered together. Gene exchanges are likely to be correlated with geographical location, which is consistent with results from previous studies [14, 24, 31–33]. Population genomics analysis, including PCA, NJ tree, and structure, reveal that red jungle fowl can be separated into two branches, possibly because there have been different venues for domestication [17, 34, 35]. The LD decay analysis showed that XBC had the highest attenuation rate among the breeds tested, suggesting that XBC has been more domesticated and subjected to higher selection intensity than other chickens. This is in accordance with our expectations, since XBC has actually undergone a relative stronger artificial selection program than other breeds. Analysis of migration patterns among different varieties revealed that XBC evolved from the red jungle fowl, and another gene flow was observed between XBC and TBC. This is because breeds such as XBC were domesticated in the middle or lower reaches of the Yellow River, and then brought to many other regions by humans [36]. Some traits such as cockfighting obviously suffered positive selection, so it is reasonable that gene flow appeared between XGF and LX. Additionally, gene flow between chickens that were geographical neighbors has been proven. Obviously, the role of human activities cannot be ignored in the domestication history of native breeds. Human cultural exchange, trade, and migration have also promoted the domestication and spread of chickens.

Because the chicken is the most common domesticated animal in the world, their domestication has always been a topic of great interest in archeology and evolutionary biology. In 2014, Chinese and foreign scholars concluded that Northern China was a domestication center for chickens, dating back to the early Holocene, approximately 10,000 years ago [17]. However, this conclusion has been contested by other researchers [37, 38]. According to animal skeleton data and genomic information, the differentiation of chickens occurred 5.78 ka years ago. This finding is consistent with the most reliable archeological evidence [39]. Our results suggest that XBC diverged about 2.89 ka years ago, earlier than the appearance of cockfighting in China. This result also shows that human activities had a considerable influence on the domestication of chickens. Consistent with previous results, PSMC analysis revealed that the effective population of chickens fluctuated over time as the earth’s climate changed. The effective population peaked at the last glacial maximum (20–26 ka), and then decreased dramatically, as is the case for populations of other species [14, 31, 40].

Animals with distinct phenotypes in morphology, physiology, and behavior are excellent models for studying potential genetic changes and evolutionary mechanisms. Many traits in chickens, such as comb mass [41], aggressive behavior [42], reproductive performance [43], plateau adaptability [23], and vision [18] were influenced by selective sweeps. These studies do not include many other distinctive phenotypes found in various chicken breeds, such as black skin caused by pigmentation. Multiple genes affect skin and shank color. Many genes related to coat and skin color, such as *SLC45A2*, *SLC24A2*, *SLMO2*, *ATP5e*, *CDKN2A*, *GRAMD3*, *FGF10*, and *EDN3*, have been found in genomic regions under selective sweep [44–49].

Variations at the nucleotide level can affect gene transcription and the evolution of an organism. In order to better study the formation of melanin, we hybridized an exogenous chicken breed with XBC and collected F2 full-sibs with black and yellow dorsal skin. We used these individuals for high-throughput RNA-Seq and identified candidate genes for melanin deposition. We found that *SLC45A2, SLMO2, ATP5e*, and *EDN3* were differentially expressed between the black and yellow dorsal skin types.

A large number of studies have established that the synthesis of melanin begins with the oxidation of tyrosine by tyrosinase, followed by a series of complex reactions. This process is regulated by a variety of signal molecules that affect the deposition and distribution of melanin in different animals. Membrane-associated transporter *SLC45A2* is a member of the free vector family of SLC45A (solute carrier family 45) [50]. It is a transporter that regulates melanin synthesis and plays an important part in melanin production [51]. Mutations in *SLC45A2* can affect the processing of tyrosinase, causing albinism in human eyes and skin [52]. *SLC45A2* is differentially expressed in the skins of 1-month-old grey and white Lander geese [53]. In agreement with these studies, we found that *SLC45A2* is differentially expressed in chicken skins of different color. The results suggest that the differential expression of *SLC45A2* affects the deposition of melanin in black skin of the XBC, although the specific regulatory mechanism remained unknown.

Recent research has shown that the Fm phenotype (associated with the Fm region) is related to domestication and has been intensely artificially selected, with about 1.5-folder blacker level [48]. A previous study found that an inverted duplication affecting *EDN3*, a key gene in dermal melanin located on chromosome 20 in the Fm region, caused excessive accumulation of black pigment in chickens [54, 55]. We also detected an inversion at 10.97–11.52 Mb and a duplication at 10.74–12.55 Mb in *EDN3*, which overlaps the Fm region. *EDN3* promotes the survival, proliferation, and differentiation of melanocytes [56, 57]. Differences in *EDN3* copy number correspond with black and white pigmentation of chicken skin [58]. *EDN3* is also the most likely candidate gene for coloration phenotypes in other domesticated animals, such as sheep [59], cats [60], and cattle [61]. The expression of lncRNA has been breed- and tissue- specific. In our study, no *lnc-TMEM184C* found by Hong et al. [62].

Green shanks are another typical characteristic of Xichuan black-bone chickens. *GRAMD3* is highly expressed in human retinal pigment epithelial cells, and might be involved in the synthesis or transport of melanin. A mutation in the noncoding region of exon 4 of *GRAMD3* may be linked to shank color in chickens [63]. Mutations in the flanking region of *GRAMD3* cause abnormal expression of the gene in green shank [64]. Further research is needed to understand how *GRAMD*3 affects the shank color.

Our results suggest that *FADS6*, *LEPR*, *LPL*, *MCHR1*, *PLIN2*, and *PYROXD1* might be associated with fatty acid-related pathways. Fatty acid desaturase 6 (*FADS6*) is related to fatty acid composition (FAC) in Hanwoo Cattle [65]. *LPL* produces fatty acids and monoglycerides for tissue use or storage. *LEPR* is an important mediator for leptin function. Mutations in the *LEPR* gene are associated with obesity in humans and fat deposition in animals such as cattle and pigs [66–68]. Most notably, *MCHR1*, when it is expressed in the hypothalamic ventromedial nucleus and paraventricular nucleus in the brain, is related to feeding behavior [69, 70]. When *MCHR1* is distributed in the peripheral tissues of mice, it affects the synthesis of fat and is related to obesity [71, 72]. There are highly specific autoantibodies against *MCHR1* in vitiligo patients that play an important role in the pathogenesis of vitiligo autoimmunity [73]. When *MCHR1* is highly expressed in the dorsal skin of *Misgurnus anguillicaudatus* (pond loach), it helps the formation of dark spots. We suggest that *MCHR1* might play an important role in the meat quality and pigmentation in XBC.

*SLCO1A2*, *SLCO1B1* and *SLCO1C1* function in pigment transportation in poultry eggshells. *SLCO2B1* is a member of the OATP gene family. The protein encoded by *SLCO2B1* might be related to biliverdin transport [74]. Brown shell traits are closely related to protoporphyrin transportation in the eggshell gland, a process that is regulated by OATP gene family members such as *SLCO1A2* and *SLCO1C1* [75].

## Conclusions

In summary, the evolutionary history, molecular phylogeny, and selection evidence of several chicken breeds were explored in this study. The results provide new insight into Xichuan black-bone chickens and their unique qualities.

## Methods

### Sample collection and sequencing

Whole blood samples from five 30-week-old female Xichuan black-bone chickens were collected from Xichuan County (32°56′11″N, 111°24′46″E) in Henan Province, which come from different conservation populations and have no genetic relationship. The pentobarbital was used for euthanization by intraperitoneal injection at a dose of 40 mg/kg of body weight according our previous study [76]. We made all efforts to decrease animal suffering. Genomic DNA was isolated using a standard phenol/chloroform extraction method. Briefly, library construction involved the following steps: DNA shearing, purification, end-repairing, ligating adaptors, size selection, and DNA amplification. The libraries were sequenced using the Illumina HiSeq 4000 platform (PE300). Sequencing and base calling were performed using the manufacturer’s protocols to generate primary read data.

Sequence data for 24 individuals from other chicken breeds was obtained from published dataset (Supplementary Table S1) [23, 44]. The downloaded data were generated using Illumina HiSeq 2000 technology with 18X coverage per sample (Supplementary Table S2). Combining our data with the downloaded data, there were 29 chickens in total, including 5 XBC, 5 Tibetan chickens (TBC) originating from Tibet, 5 Xishuangbanna game fowl (XGF) originating from Dai Autonomous Prefecture of Xishuangbanna of Yunnan Province (raised for cockfighting), 5 Yunnan Village chickens (YVC, raised for food), 5 Red Junglefowl (RJF) originating from Yunnan Province, and one Dongxiang blue-eggshell chicken (DX) and one Silkie chicken (SK), both originating from Jiangxi Province, one Luxi fighting chicken (LX) originating from Shandong Province, and one Wenchang chicken (WC) originating from Hainan Province. The data were used to analyze the evolutionary history of Xichuan black-bone chickens and other populations (Fig. 1a). Breed origins were as described by the Animal Genetic Resources in China for Poultry.

### Quality control processing and variant calling

To ensure high-quality data, the raw sequencing data for all breeds were filtered to remove adaptors, trim low-quality and unidentified nucleotides (Ns) at the 5′ and 3′ ends, trim nucleotides having an average quality score less than 30 within a window of 4 nucleotides, and removing reads containing more than 10% Ns. After these steps, reads with lengths less than 30 bp were removed. High-quality reads were aligned to the chicken reference genome (version: galGal5) using BWA-MEM (version: 0.7.12) [77]. Sequence variants were identified using a GATK Best Practices workflow to identify single nucleotide polymorphisms (SNPs) and insertion/deletion sequences (InDels) [78–80]. Detection of Structural variations (SVs) and copy number variants (CNVs) was performed using BreakDancer [81] and CNVnator [82]. Finally, gene-based annotation of the variants was accomplished using SnpEff [83], for which the corresponding chicken gene annotation file was downloaded from Ensembl. The distribution of InDels, SNPs, CNVs, and SVs was visualized using Circos [84] and arranged in chromosomal order. Whole-genome identity score (IS) were calculated with SNP frequencies [85]. To compare the Xichuan black-bone chicken with the other breeds, we adjusted the sequencing data for the Xichuan black-bone chicken so that the average sequencing depth was similar across breeds.

### Population structure and linkage disequilibrium analysis

To reduce noise due to linkage disequilibrium (LD), SNPs with high pair-wise R^2^ values (R^2^ > 0.2) were pruned from the dataset using PLINK (v1.90, arguments: --indep-pairwise 50 5 0.2) [86]. The neighbor-joining tree (NJ) was constructed with Nei’s genetic distances using the phylogeny program MEGA v7.0 [87, 88]. Principal component analysis (PCA) was performed using Genome-wide Complex Trait Analysis (GCTA) [89] and visualized with R package (ggplot2). The population structure was investigated using the supervised ADMIXTURE program (Version 1.3) [90] using the maximum likelihood method. We predefined K, the number of genetic clusters (ranging from K = 2 to K = 10). LD for five breeds was calculated on the basis of the correlation coefficient R^2^ statistics of two loci using PopLDdecay [91].

### Gene flow, estimation of divergence time, and demographic history

TreeMix [27] was used to construct a population phylogenetic tree for gene flow analysis of the 9 breeds. The divergence time between XBC and other groups was estimated using Beast2 and TreeAnnotator [92]. The alignment result was converted into Nexus file format and used as the input for Beast2. Parameters were set as follows: Partition, Unlink; Site Model, HYK; Gamma Category Count, 4; Clock model, Strict; Priors tree prior, Yule Model; MCMC, 10,000,000; Tracelog, Screenlog and treelog.t:tree, default. A tree file was one of the outputs generated by Beast2. The tree file was used as input to TreeAnnotator. We used a pairwise sequentially Markovian coalescent (PSMC) model [93] to detect changes in the effective ancestral population sizes of the XBC population at a high level of detail. We set g = 1 and a mutation rate of 1.91 × 10^− 9^ per generation to estimate the distribution over time.

### Selective sweep analysis

To define candidate regions that had undergone directional selection in the XBC, we calculated the population differentiation index (*Fst*) between black-skin chickens (XBC, DX, SK) and non-black-skin chickens (TBC, XGF, RJF, YVC, LX, WC), as described by Weir & Cockerham [94]. We calculated the average *Fst* value in 10 kb sliding windows with a 5 kb sliding step between all black and all non-black populations using VCFtools (v0.1.13) [95]. The fixation index (*Fst*) measures the genetic differentiation between two populations. For each SNP, F*st* is defined as:

${F}_{ST}=1-\frac{p_1\left(1-{p}_1\right)+{p}_2\left(1-{p}_2\right)}{\left({p}_1+{p}_2\right)\left[1-\left({p}_1+{p}_2\right)/2\right]}$, where *p*_1_ and *p*_2_ are the frequencies of an allele in all black and non-black populations, respectively.

Nucleotide diversity (pi) was also estimated for each sliding window across all sampled populations. The pi primarily reflects the nucleotide difference. The top 5% of *Fst* values were retained as candidate sweeps. The sweeps also exhibited large differences in pi values between black and non-black populations. Finally, we performed a functional enrichment analysis of the candidate genes, using Gene Ontology (GO) categories and Kyoto Encyclopedia of Genes and Genomes (KEGG) pathways. The analysis was conducted with the Database for kobas3.0 (http://kobas.cbi.pku.edu.cn/kobas3).

### RNA-Seq and data processing

To determine whether novel allelic variants located in the top 5% of *Fst* regions of the genome affect gene expression, we utilized RNA-Seq to construct six cDNA libraries from black (BS) and yellow (YS) dorsal skin of a Xichuan black-bone chickens hybrid population. The hybrid F2 population was obtained by crossing the Gushi chickens with Xichuan black-bone chickens. All the chickens were raised in cages under the same environment with ad libitum conditions. Full-sibs with different skin colors of 1-day-old female chickens were collected for RNA-seq. RNA-seq libraries with insert sizes of approximately 300 bp were prepared using the Illumina standard RNA-seq library preparation pipeline and sequenced on the Illumina HiSeq 4000 platform.

Adaptor sequences and low-quality sequence reads were removed from the data sets. Clean reads were then mapped to the reference genome sequence (https://www.ncbi.nlm.nih.gov/genome/?term=chicken). Differentially expressed genes were detected with edgeR (v3.6) [96, 97] using a threshold of |log2 (fold change) | ≥1 and adjusted *p* < 0.05.

### Quantitative RT-PCR and data analysis

To verify the accuracy of the data obtained from RNA-seq, the results were confirmed by qRT-PCR. Six gene-specific qRT-PCR primers were designed using the Primers-BLAST online program at NCBI and synthesized by Shenggong Biological Engineering (Wuhan, China) Limited by Share Ltd. Primer sequences are shown in Supplementary Table S3. All qPCR was conducted using a LightCycler® 96 Real-Time PCR system (Roche Applied Science). Each sample was assayed three times. Chicken *β-actin* gene was used as a reference gene for the qPCR assays. Relative expression was calculated using the 2^-ΔΔCt^ method [98].

## Supplementary information

**Additional file 1: Supplementary Figure S1.** Statistics of genetic variants in Xichuan black-bone chickens.

**Additional file 2: Supplementary Figure S2.** Gene flow analysis of 29 chickens. Graphs were generated for one (A), two (B), three (C), and four (D) migration events. Migration events are shown by colored arrows. Migration weight is represented by color intensity.

**Additional file 3: Supplementary Figure S3.** GO analysis of candidate genes under selection in Xichuan black-bone chickens.

**Additional file 4: Supplementary Figure S4.** Verification of RNA-seq results by qRT-PCR.

**Additional file 5: Supplementary Table S1.** Data sources for chickens used in this study.

**Additional file 6: Supplementary Table S2.** Statistical summary for analysis of downloaded chicken sequence data (24 libraries).

**Additional file 7: Supplementary Table S3.** Primers used for qRT-PCR in this study.

**Additional file 8: Supplementary Table S4.** Statistical summary of sequencing data obtained for 5 XBC libraries by Illumina deep sequencing.

**Additional file 9: Supplementary Table S5.** LD decay.

**Additional file 10: Supplementary Table S6.** Time of divergence between populations.

**Additional file 11: Supplementary Table S7.** Gene annotation of genomic regions with strong selective sweep signals in Xichuan black-bone chickens.

**Additional file 12: Supplementary Table S8.** Difference in expression for dorsal skin between black skin (BS) and yellow skin (YS) in Xichuan black-bone chickens by RNA-Seq.

**Additional file 13: Supplementary Table S9.** Coding potential of *TCONS_00054154*.

**Additional file 14: Supplementary Table S10.** Bioinformatics analysis of *TCONS_00054154*

## Data Availability

Data for the 5 Xichuan black-bone chickens used in the whole-genome resequencing analysis, and for the 6 Xichuan black-bone chickens used in the RNA-Seq analysis are accessible at NCBI under BioProject accession numbers PRJNA642410 and PRJNA418694, respectively. Illumina paired-end sequences for the other 24 chickens used in this study were downloaded from NCBI with SRA accession numbers SRP034930 and SRP040477. Chicken reference genome sequence was downloaded from NCBI (https://www.ncbi.nlm.nih.gov/genome/?term=chicken). chicken gene annotation file was downloaded from Ensemble (ftp://ftp.ensembl.org/pub/release-100/gff3/gallus_gallus).

## Abbreviations

XBC: Xichuan black-bone chicken
TBC: Tibetan chickens
WC: Wenchang chicken
SK: Silkie chicken
DX: Dongxiang blue-eggshell chicken
LX: Luxi fighting chicken
BS: Black skin
YS: Yellow dorsal skin
InDels: Insertion/deletion polymorphisms
SNPs: Single nucleotide polymorphisms
CNVs: Copy numbervariations
SVs: Structural variations
IS: Identity score
LD: Linkage disequilibriu
NJ: Neighbor-joining
PCA: Principal component analysis
GCTA: Genome-wide Complex Trait Analysis
GO: Gene Ontology
KEGG: Kyoto Encyclopedia of Genes and Genomes
