# Supplementary figures and images for: Breeding history and candidate genes responsible for black skin of Xichuan black-bone chicken
Source: BMC Genomics. 2020 Jul 23;21:511. doi: 10.1186/s12864-020-06900-8 (PMC7376702; doi:10.1186/s12864-020-06900-8)

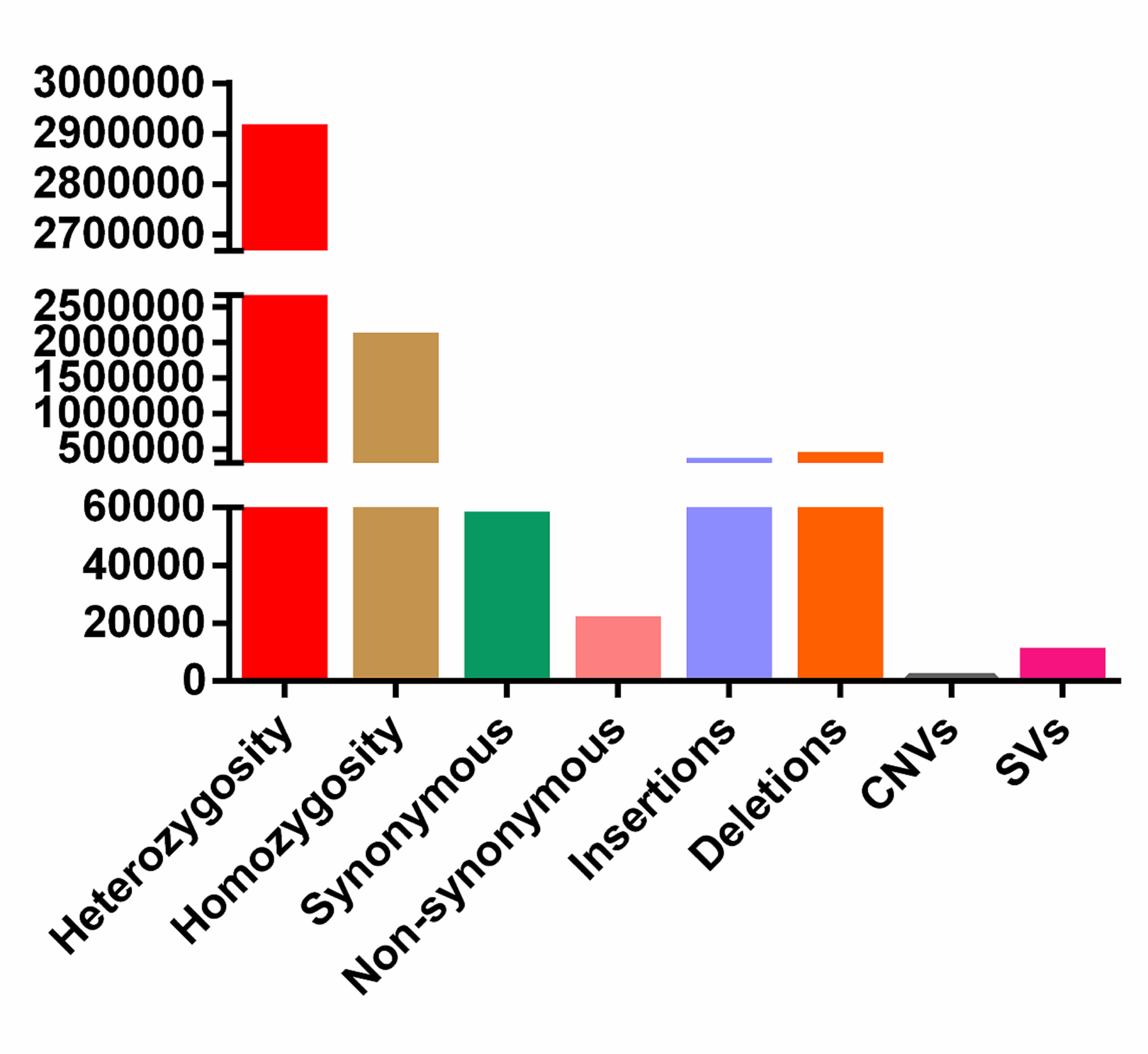

Supplement: Supplementary file 1 — Additional file 1: Supplementary Figure S1. Statistics of genetic variants in Xichuan black-bone chickens. [file 12864_2020_6900_MOESM1_ESM.tif]

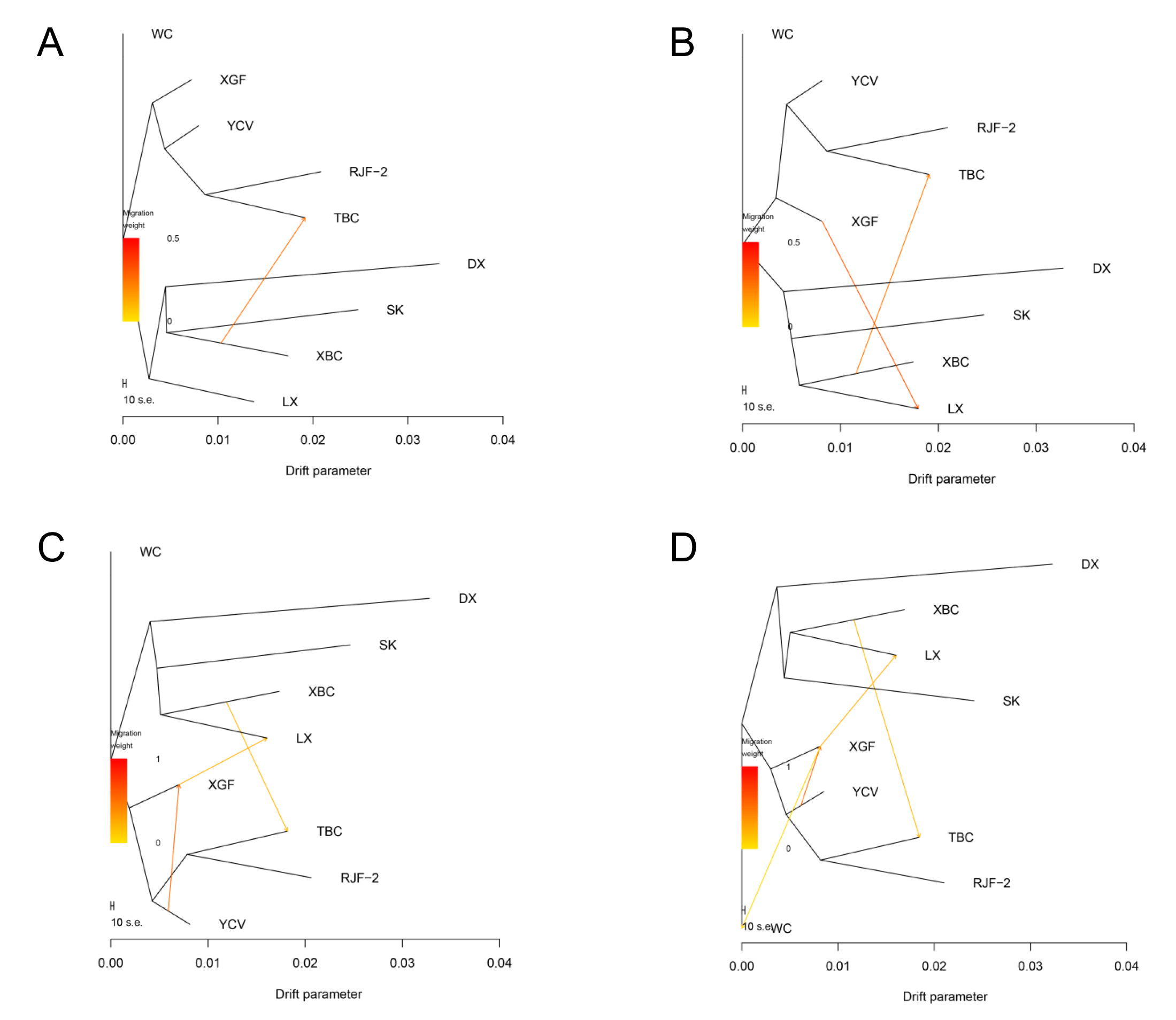

Supplement: Supplementary file 2 — Additional file 2: Supplementary Figure S2. Gene flow analysis of 29 chickens. Graphs were generated for one (A), two (B), three (C), and four (D) migration events. Migration events are shown by colored arrows. Migration weight is represented by color intensity. [file 12864_2020_6900_MOESM2_ESM.tif]

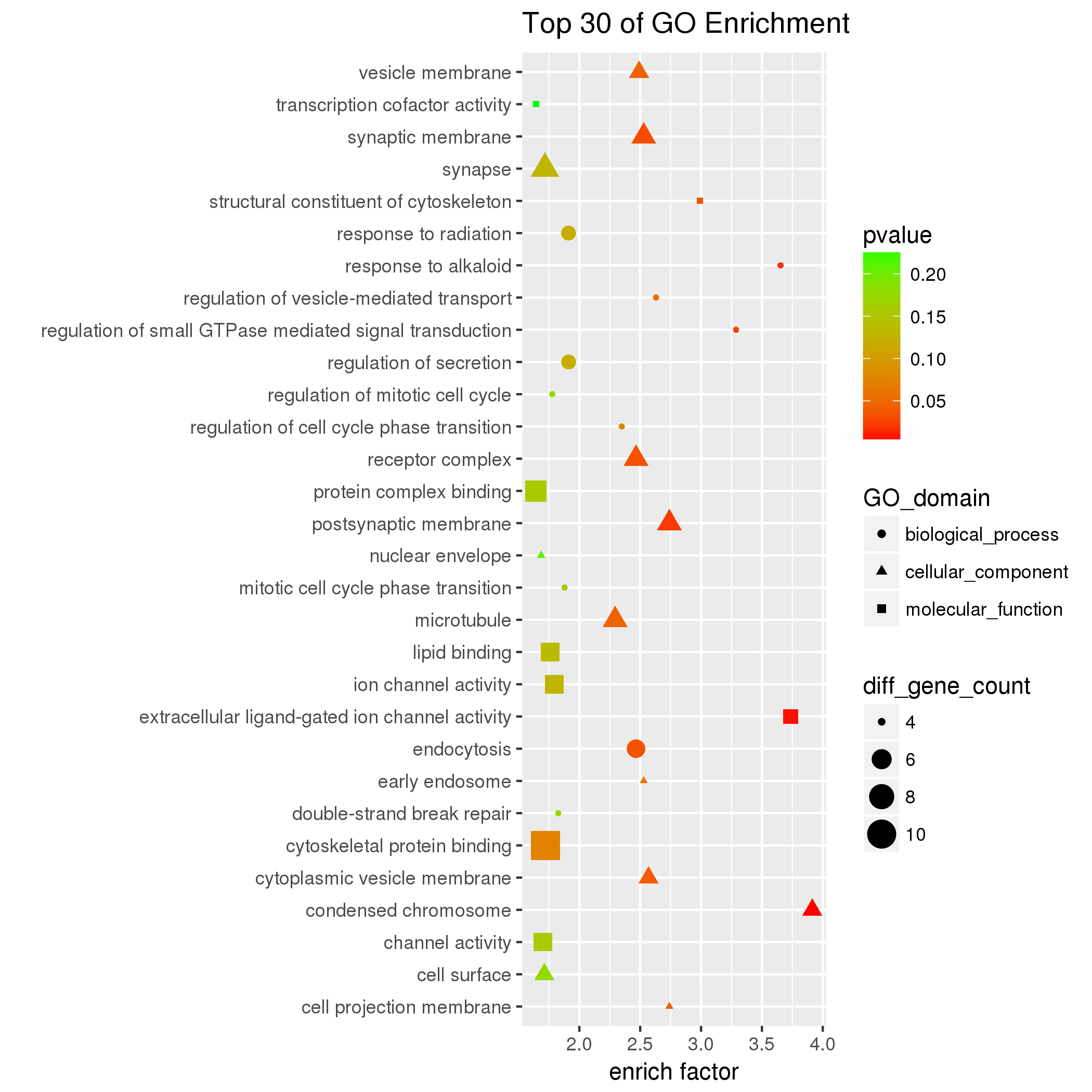

Supplement: Supplementary file 3 — Additional file 3: Supplementary Figure S3. GO analysis of candidate genes under selection in Xichuan black-bone chickens. [file 12864_2020_6900_MOESM3_ESM.tif]
