# Supplementary material for: Breeding history and candidate genes responsible for black skin of Xichuan black-bone chicken
Source: BMC Genomics. 2020 Jul 23;21:511. doi: 10.1186/s12864-020-06900-8 (PMC7376702; doi:10.1186/s12864-020-06900-8)
Supplement: Supplementary file 7 — Additional file 7: Supplementary Table S3. Primers used for qRT-PCR in this study. [file 12864_2020_6900_MOESM7_ESM.pdf]

Supplementary Table S3 Primers used for qRT-PCR in this study.

Gene ID	Product siz	Primer sequences(5'-3')	Primer sequences(3'-5')
ATP5E	249	TGAACAGTCTTCCAGCGTGC	CAGGTGCACAGAAGTTCGTC
SLMO2	163	GAAGCCTTCAGGCAATGCA[illegible]	TCGCACGTTACACAGAGGTC
EDN3	179	TCAACACCCCAGAGAGGAC[illegible]	GCAAACGTTAAACCTTCTTCTGTC
SLC45A2	202	AGGGCTCTTGGTCAGTGTTG	AAGAGGGGAGGGTGTACCAG
MSLNL	152	GCCACAGAAATGAACCCCC[illegible]	GTGGAAGAGGTGCCAAGAGG
HPX	141	CCCGTGATCTCCGTGACTAC	CATAGATGCGGCCACTGTCA
β-actin	216	TGCCAGGGTACATTGTGGTA	TGCGTGACATCAAGGAGAAG

Tm
60 ℃
60 ℃
60 ℃
60 ℃
60 ℃
60 ℃
60 ℃
