# Supplementary material for: Breeding history and candidate genes responsible for black skin of Xichuan black-bone chicken
Source: BMC Genomics. 2020 Jul 23;21:511. doi: 10.1186/s12864-020-06900-8 (PMC7376702; doi:10.1186/s12864-020-06900-8)
Supplement: Supplementary file 9 — Additional file 9: Supplementary Table S5. LD decay. [file 12864_2020_6900_MOESM9_ESM.pdf]

Supplementary table S5 linkage disequilibrium (LD) decay

popid.i.	decay05	decay01	maxld	decay05.r	decay01.r	maxld.r
TBC	271	294482	0.5564	0.5564	0.20006	0.594704277
XGF	553	264949	0.5309	0.5309	0.20008	0.596828545
RJF-2	7970	256292	0.6124	0.6124	0.33334	0.668667158
YCV	357	289692	0.5362	0.5362	0.16712	0.577352688
XBC	903	296114	0.5988	0.5988	0.20068	0.602580805
