# Supplementary material for: Breeding history and candidate genes responsible for black skin of Xichuan black-bone chicken
Source: BMC Genomics. 2020 Jul 23;21:511. doi: 10.1186/s12864-020-06900-8 (PMC7376702; doi:10.1186/s12864-020-06900-8)
Supplement: Supplementary file 10 — Additional file 10: Supplementary Table S6. Time of divergence between populations. [file 12864_2020_6900_MOESM10_ESM.pdf]

Supplementary tables S6 Time of divergence between populations

```
#NEXUS

Begin taxa;
	Dimensions ntax=5;
		Taxlabels
			RJF-2
			TBC
			XBC
			XGF
			YCV
			;
End;
Begin trees;
	Translate
		   1 RJF-2,
		   2 TBC,
		   3 XBC,
		   4 XGF,
		   5 YCV
;
tree TREE1 = (1[&height=0.0,length=5.809498571010125,length_95%_HPD={5.079986915510172,6.
End;
```

680439342746728},length_median=5.743394273341674,length_range={4.709457354417239,6.798823

38878031166}]:5.809498571010125,(2[&height=0.0,length=4.855528725504431,length_95%_HPD={4.1

12267012445876,5.599282241623246},length_median=4.806924332251451,length_range={4.1226701[illegible]

2445876,5.962910685266433}]:4.855528725504431,(3[&height=0.0,length=4.150209961038726,len[illegible]

gth_95%_HPD={3.4203286682595704,4.883953759601065},length_median=4.140702155650646,length_

_range={3.4203286682595704,5.2382501821371195}]:4.183043443918512,(4[&height=0.0,length=3.

.6699307791470765,length_95%_HPD={3.1303558656119126,4.471000822968752},length_median=3.5[illegible]

65589388138941, length_range={2.948054355278409,4.534439789331132}]:3.631369858746698,5[&h

eight=0.0,length=3.63709729626729,length_95%_HPD={3.1303558656119126,4.41597942843575},le

ngth_median=3.5640035405196313,length_range={2.948054355278409,4.534439789331132}]:3.6313

69858746698)[&height=3.631369858746698,height_
