# Supplementary material for: Breeding history and candidate genes responsible for black skin of Xichuan black-bone chicken
Source: BMC Genomics. 2020 Jul 23;21:511. doi: 10.1186/s12864-020-06900-8 (PMC7376702; doi:10.1186/s12864-020-06900-8)

**Supplementary table S10 Bioinformatics analysis of TCONS_00054154**

TCONS_00054154

AGTAGCAGTACAGTGGATTAACTTGTTTCACGTTACTTGCAAGGTGTTTTAAGAAGATCTGTCTGGTTTGTAACTTCTCAGTGCTGTGTAGTTGTCTCGATTCCATGCACCCACGTGCACTGTAAGTTTTTGCTAAAGACTTACGTTTGTGCTCTGTAAATCCTGGTGATAGCCTAACATTTCAGTGTTCTGAAAGATGCCCCTATTTGCTATCTGGAATAACAAAAACTGGGGCCATGGAAAAGGGAAAATTCTTAAACTTATTGTAACGCTGCAATGACTTGTGTCTATGTGTATACTAAAAACATACTTTTCTTTTAATGACTGAAGTCTAAGAAAACAAAAGGCTTTGTTTATCCAAAAATATTTCTTCTTGCTAACAATGGGTGACAGCACCTGTTTGTTCACATAGCAGTGAGCAACTGATTTACAGCTTATTGTGGGAGGGGGTGTGGAATTAACCACAATTGCCTACAACACTGCTGTTTTGTCTGTTGTGGTCTGCATTTGCAGTTAAGAGCATACAGTTGAGTTTCCTTCCAGTAAGGTAAGGCTAAACGCCCACAGTCTGCACTCCCTTTGCACTGACAGACACTGGGTTCAAAGGAAAAAATAAGCATCCTGTTGAGGCTCAAAAGTGAACAGAGGAACTGAAGAATGCTGATCATACAAGTCTGTCTGAAAGCTCTGAGTCATTTTAGAATGCCCATGTTCTTCAGGGTGTGAAGTTTGTTCCTGCACTTGTGCAGGACTTGTAGAGCAATCTAATAGACACGTTGTGAAATTAGAAGTAAAGAAACTGTACTACTGTAATATGATGGAAAATGTAGCAATAACAGCAGGGCAGAAAAGCCTGTAGCTGCAGCAAATGAGAACAAGCCCAAATGAAGCAGGTATGGATGCATAAACAAAGGAAGAAAGTTTGCTTACCTTCAGAAGGCCTCAGGCACACAAGGATCTCCGGTAAGATCCCCTCACATTAAATGAAGGCTGGGAAGGGGTGGATCCTGGCTGCAGCCCTTCCAGTCACTCAGATGCATTGCTTACACCTGAGCTCCCCTGGGTTGGCCCTGCCTTCCCAACATCTGCTCCATCACTGCTTCAGGCCCTGACTCAGCATTCCTGCTGAATTCTTCTCTGCAGATCCTGGGCTCCTCCAGAGACTATTTATTCCCAGCTGAGAACTTGGCAGACCAGTCTGTTGGAAGTCGTGTTGGTACTGGCTTTTCTTTGTACTGCAGTTTTCCTTCTGTACAATTATTTTTTCTTCCCTTTATAGTGAGAGGCTGGGTTTGACTTGAAGCCGCCACGTACATCTGTACATTTTTTATTGTAATGTCTGTACATAACCAGTAAGCAGCCCAGTGTCTGAATATCCCATGGTACTTTATGACCACGTGTAATGCGTTTCTGGCACGTTACAGGCTCTGGAACCACTGCAGGCTACGCACGGGCCCTTTTCTGGATCCCTGAAGCAGGGCCCAACTGGCCGCATCTGGAAGTGGAGCAGAGGCCCTCCCGAACCACGTCCTACTCTGGGCAAACCACGCAAAGAACTGCCGAGGCGGAGCCATGCTGAACACCCACCGCAACCGCCGCGGGCAAGGCGG

NCBI blast


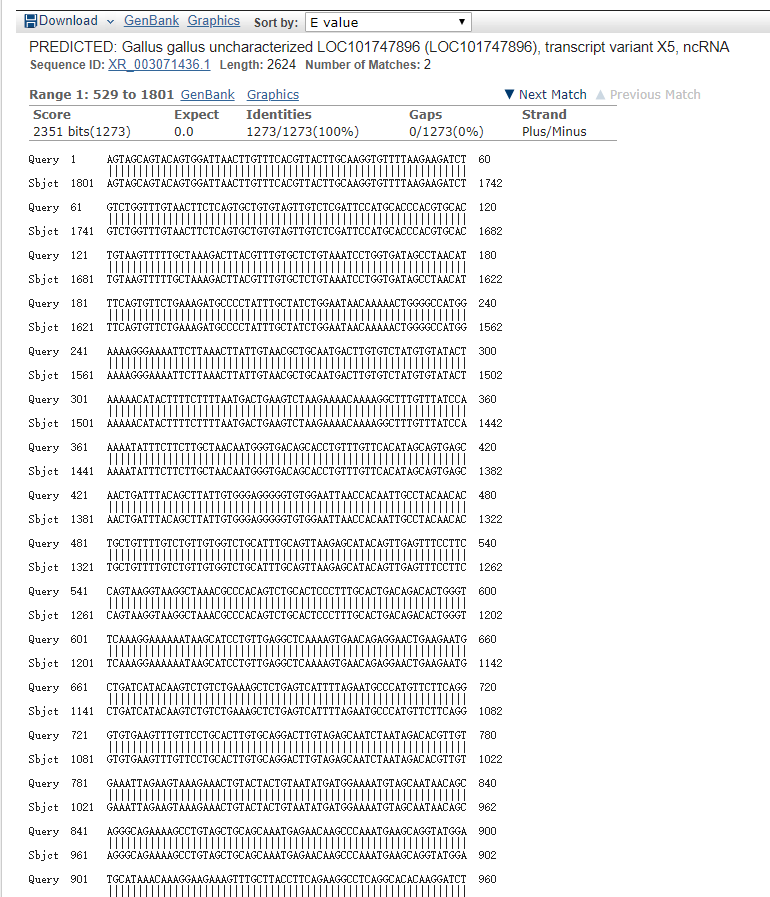


Chromosomal location


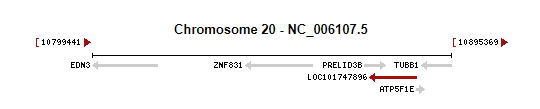

Supplement: Supplementary file 14 — Additional file 14: Supplementary Table S10. Bioinformatics analysis of TCONS_00054154 [file 12864_2020_6900_MOESM14_ESM.docx]
